# The impact of age-specific childhood body-mass index on adult cardiometabolic traits: a Mendelian randomization study

**DOI:** 10.3389/fendo.2023.1159547

**Published:** 2024-01-15

**Authors:** Jun Yang, Yalan Kuang, Xiaoyan Yang, Chunyang Li, Mei Qi, Ping Fu, Xiaoxi Zeng

**Affiliations:** ^1^Department of Mathematics, Med-X Center for Informatics, Sichuan University, Chengdu, China; ^2^West China Biomedical Big Data Center, West China Hospital, Chengdu, China; ^3^Division of Nephrology, The Second People’s Hospital of Tibet Autonomous Region, Lhasa, China; ^4^Division of Nephrology, Kidney Research Institute, West China Hospital, Sichuan University, Chengdu, China

**Keywords:** childhood, body mass index, cardiometabolic traits, sex disparity, genome-wide association study, Mendelian randomization

## Abstract

**Objective:**

To evaluate the causal relationship between childhood body-mass index (BMI) at different ages and adult cardiometabolic traits.

**Methods:**

We retrieved genetic instrument variables (IVs) for exposures (standardized BMI at newborn, infant, toddler and late childhood), cardiometabolic traits and potential confounders or mediators (adult BMI, SHBG, testosterone and age at menarche) from the corresponding genome-wide association analysis. We performed univariate and multivariable Mendelian randomization (MR) to dissect associations between age-specific childhood BMI and adult cardiometabolic outcomes. Odds ratio was used to present the direction of the causal association.

**Results:**

In univariate MR, higher newborn BMI was causally associated with reduced risk for type 2 diabetes in women. Late childhood BMI was associated with increased risk for female diabetes and coronary artery disease (CAD), myocardial infarction (MI), and chronic kidney disease (CKD) in general population. Among these associations, only association between late childhood BMI with MI remained significant after adjusting for adult male BMI and sex hormones, (OR = 1.120, 95% CI 1.023-1.226, p = 0.014). Besides, in multivariable MR, we found evidence for causal association between newborn BMI with reduced risk for CAD (OR = 0.862, 95% CI 0.751-0.989, p = 0.034) and MI (OR = 0.864, 95% CI 0.752-0.991, p = 0.037) in men. No obvious impact of infant or toddler BMI was identified on the above-mentioned diseases. For continuous cardiometabolic traits, in all age epochs except infant, higher BMI was associated with increased level of fasting glucose in women.

**Conclusion:**

BMI at birth and late childhood exerts different impact on adult cardiometabolic diseases, while BMI at infant and toddler ages is not causally associated with these outcomes. The effect of childhood BMI may be influenced by sex disparities.

## 1 Introduction

Early increase in body mass index (BMI) is associated with risk of obesity in adulthood, a major public health issue worldwide, and the many complications that follow. Some studies reported that high childhood BMI is associated with cardiometabolic diseases in adulthood, such as coronary artery disease (CAD), myocardial infarction (MI), chronic kidney disease (CKD) and diabetic mellitus (DM) (1).

However, childhood BMI has a significant change pattern with age. From birth, BMI rises rapidly until it reaches a maximum at 9 months of age and then gradually declines to a minimum around 5-6 years of age (2, 3). In addition, rapidly changing genetic landscape of childhood BMI has also been proposed that the association of common genetic variation with BMI changes rapidly during infancy and early childhood (4). Considering the significant alterations in both phenotypic and genetic variation in childhood BMI at different ages, whether childhood BMI exerts an age-specific impact on adulthood cardiometabolic traits remains to be assessed. This would provide evidence for age-specific intervention for childhood obesity for the primary prevention of adult cardiometabolic diseases. Furthermore, whether childhood BMI exerts direct and independent influence on the risk of adult cardiometabolic diseases, or is associated with these diseases through the close linkage with other causal factors (i.e. prolonged exposure to higher BMI into adulthood (5), remains controversial. For instance, the association between childhood BMI with adult trait outcomes observed in univariate Mendelian randomization (MR) analysis shrinks after accounting for adult BMI (1). Moreover, childhood BMI is associated with sex hormones (6), which are among potentially causal risk factors for adult cardiometabolic diseases. Nonetheless, the influence of sex disparity on the childhood BMI-adult traits relationship has not been fully explored.

MR is an approach that can help deal with these challenges, which uses genetic variants of the exposure as instrumental variables (IVs) to explore the causal relationship between exposure and outcome (7). Previous studies have used MR to infer causality among correlated traits (8, 9). As the genetic variant will not be influenced by the early stages of the disease process, then the estimate of the causal effect of interest will thus be immune to the reverse causation, and confounding is considerably less evident than in conventional observational studies (10).

To further validate the influence of childhood obesity on adult outcomes, and untangle the crosstalk among age-specific BMI, sex disparities and adult cardiometabolic outcomes, by using univariate and multivariable MR, we conducted the study to assess the causal influence of childhood BMI at different ages on adult cardiometabolic traits or diseases, including CAD, MI, CKD, DM, fasting glucose and fasting insulin, accounting for possible confounders or mediators including adult BMI and sex-related parameters.

## 2 Materials and methods

### 2.1 Study design

According to the American Medical Associations’ age designations (https://www.nih.gov/nih-style-guide/age) and previous research, based on genome-wide association study (GWAS) summary statistics of childhood BMI at birth, 6 weeks, 3, 6 and 8 months, 1, 1.5, 2, 3, 5, 7 and 8 years, we divided childhood into four stages (1): newborn: at birth (2); infant: 6 weeks to 8 months old (3); toddler: 1 to 3 years old; and (4) late childhood: 5 to 8 years old when the adiposity rebound was reported most likely to occur (3). Then, we conducted univariate and multivariate two-sample MR to explore the total causal effect and the potential independent causal associations between childhood BMI and cardiometabolic diseases or traits in adulthood, adjusting for adult BMI and sex-related parameters in the multivariate MR. We selected CAD, MI, DM, fasting glucose and fasting insulin as the cardiometabolic traits of interest in the current study. In addition, we included CKD (but not glomerular filtration rate or albuminuria) as a cardiometabolic outcome, similar to previous research (11).

Two-sample MR analysis could investigate the potential causal effect of exposure on outcome in a cost-efficient way as a large amount of GWAS summary data is deposited in public databases. Multivariable MR is an extension of MR which can be used to determine whether several exposures affect an outcome through the same pathway or whether the exposure have independent effect (12).

### 2.2 Selection of instrumental variables and data sources

**Figure 1:**
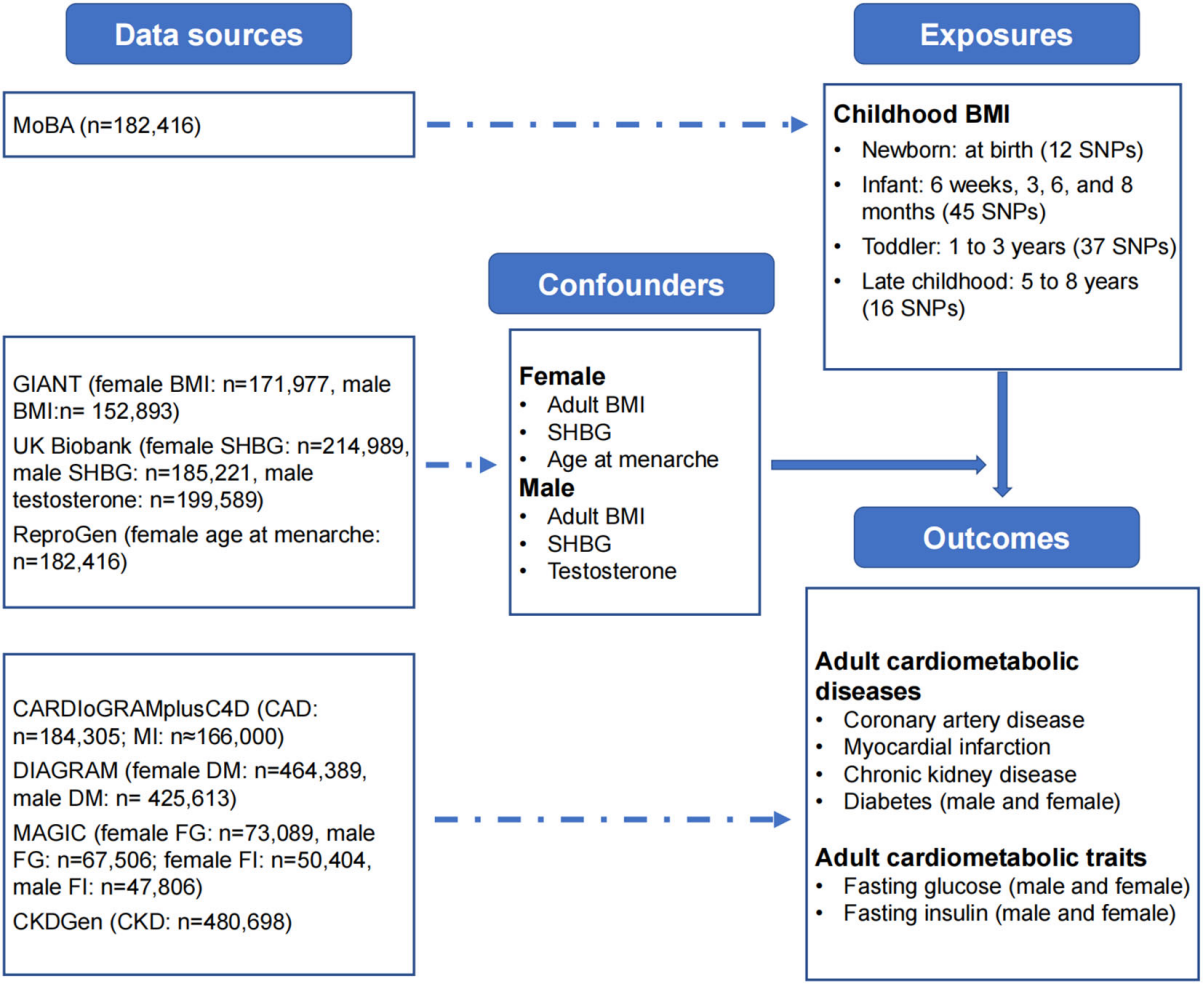
Data sources. Summary-level data sources: childhood BMI: Norwegian Mother, Father and Child Cohort Study (MoBa); adult BMI: Genetic Investigation of ANthropometric Traits (GIANT) Consortium; SHBG and testosterone: UK Biobank; age at menarche: Reproductive Genetics (ReproGen) Consortium; diabetes: Diabetes Genetics Replication and Meta-analysis (DIAGRAM) Consortium; fasting glucose and fasting insulin: Meta-Analyses of Glucose and Insulin-related Traits Consortium (MAGIC); coronary artery disease and myocardial infarction: Coronary artery Disease Genome-wide Replication and Meta-analysis (CARDIoGRAM) plus the Coronary Artery Disease (C4D) Genetics (CARDIoGRAMplusC4D) Consortium; CKD: CKDGen Consortium. BMI, body-mass index; FG, fasting glucose; FI, fasting insulin; CKD, chronic kidney disease; SHBG, sex-hormone binding globulin.

The genetic instrumental variables applied in the two-sample MR studies should satisfy three assumptions (1): associated with the exposure (2); not associated with any confounder of the exposure–outcome association; and (3) no causal pathway from instrumental variables to results except through exposure (13). To meet these three assumptions and obtain as many instrumental variables as possible, first, we selected single nucleotide polymorphisms (SNPs) which reached certain thresholds of genome-wide significance. We included SNPs at $\text{p}<5\times 10^{-8}$ for adult BMI and sex hormones. In order to ensure the inclusion of sufficient numbers of instrumental SNPs for BMI at different stages of childhood, we set a less stringent threshold of $\text{p}<5\times 10^{-7}$ (14, 15). We set the clumping $r^{2}$ cutoff at 0.01 and the clumping distance cutoff 10000 kb to remove the linkage disequilibrium, and excluded palindromic SNPs (i.e., SNPs with A/T or C/G) with intermediate allele frequency (i.e., 0.42-0.58). If a variant was significantly associated with childhood BMI at more than one time point within one age stage, we chose the association statistics from the time point with the smallest p value. The corresponding F-statistics were all greater than 10, indicating sufficient strength. Second, we restricted our analyses in individuals with European ancestry. Third, we removed SNPs that were detected to be pleiotropic and applied multivariate MR that allow inclusion of pleiotropic genetic variants to assess the independent effect of exposures. Finally, 12, 45, 37 and 16 SNPs were selected as instrumental SNPs for childhood BMI at birth, infant, toddler and late childhood (**Supplementary Table 1**; **Figure 1**). We observed 11 overlapping SNPs in the infant and toddler stages.

The full GWAS summary statistics for childhood BMI were available at the Norwegian Institute of Public Health website [Norwegian Mother, Father and Child Cohort Study (MoBa) - NIPH (fhi.no)]. BMI from 28,681 children was measured at birth, 6 weeks, 3, 6 and 8 months, and 1, 1.5, 2, 3, 5, 7 and 8 years of age (4). GWAS was conducted using linear mix-model regression on standardized, instead of directly-measured, BMI, thus, we focused on the direction of the causal association between childhood and adult outcomes in the current study. We used odds ratio (OR) greater than 1 and less than 1 to indicate positive and negative association respectively, and we considered a 95% confidence interval of OR that did not cross 1 as an indicator of statistical significance.

Summary-level GWAS statistics of adult BMI for females (ieu-a-974) and adult BMI for males (ieu-a-785) were obtained from the IEU GWAS database [IEU OpenGWAS project (mrcieu.ac.uk)], a genome-wide association study and a metabochip meta-analysis of in up to 171,977 European women and 152,893 European men (16). Instrumental variables for SHBG for females (ieu-b-4870), SHBG for males (ieu-b-4871), and testosterone for males (ieu-b-4865) were also available from the IEU GWAS database. We did not use estrogen in this study, since the large GWAS were mainly based on the UK Biobank in which significant proportion of female participants were menopausal, thus with low level of estrogen. Instead, we used age at menarche as a proxy for estrogen in female (17). GWAS summary statistics for age at menarche (ieu-a-1095) were obtained from 182,416 women of European descent from 57 studies (18).

Sex-specific GWAS summary statistics were obtained from the Diabetes Genetics Replication and Meta-analysis (DIAGRAM) Consortium [DIAGRAM Consortium (diagram-consortium.org)] for DM (n = 464,389 for females, n = 425,613 for males) (19), and the Meta-Analyses of Glucose and Insulin-related Traits Consortium (MAGIC) [Magic Investigators - Home Page] for fasting glucose (n = 73,089 for females, n = 67,506 for males) and fasting insulin (n = 50,404 for females, n = 47,806 for males) (20). For the remaining traits that sex-specific IVs were not available, instrumental SNPs from the general population were used. We obtained from the Coronary Artery Disease Genome-wide Replication and Meta-analysis (CARDIoGRAM) plus the Coronary Artery Disease (C4D) Genetics (CARDIoGRAMplusC4D) Consortium [CARDIoGRAMplusC4D - CARDIoGRAMplusC4D Consortium] for CAD (n = 184,305) and MI (n ≈ 166,000) (21), and the CKDGen Consortium [CKDGEN Meta-Analysis datasets (uni-freiburg.de)] for CKD (n = 480,698).

### 2.3 Mendelian randomization and sensitivity analyses

MR was used in this study to estimate the causal effect of childhood BMI on adult cardiometabolic diseases or traits. First, we harmonized the GWAS summary data of both childhood BMI and adult cardiometabolic diseases or traits by matching the genetic IVs. For the SNP that did not present in the GWAS of adult cardiometabolic outcomes, we used SNPs that had an LD $R^{2}>0.8$ as a proxy.

For MR analyses, the inverse-variance weighted (IVW) method was conducted as the main analysis of MR. Random-effect IVW model (IVW-RE) was applied if there was significant heterogeneity, otherwise fixed-effect model (IVW-FE) was used. We used the Cochran-Q test to measure the between-SNP heterogeneity. To confirm the robustness of the results, we also performed several sensitivity analyses, including MR-Egger regression, weighted median method, and weighted mode method. The MR-Egger method evaluated the directional pleiotropy of the IVs and the slope of the MR-Egger regression provided causal estimates corrected by pleiotropy (22). Weighted median and mode methods are robust to outliers. Additionally, MR-PRESSO analysis was performed to detect outliers defined p value <1 and to calculate outliers-corrected causal effect (23).

To evaluate the independent effect of childhood BMI on adult cardiometabolic diseases or traits, we performed multivariable MR to adjust for potential pleiotropic factors, including adult BMI and sex-related parameters using GWAS summary statistics obtained in both males and females. In addition, for DM, fasting glucose and fasting insulin in which genetic instruments for females and males were available, we evaluated the independent influence of age-specific childhood BMI in women adjusting for adult BMI, SHBG and age at menarche; and in men adjusting for adult BMI, SHBG and testosterone.

Given the involvement of 9 different causal associations using univariable MR analysis, a Bonferroni corrected P value of 0.006 (0.05/9) was established as the threshold for statistical significance. A p value <0.05 was considered suggestive evidence of a causal association. Meanwhile, we interpreted the statistical analysis results not only based on p value, but also effect value and confidence interval (24). All analyses were conducted with TwoSampleMR and MRPRESSO packages of R software.

## 3 Results

### 3.1 Univariate MR on the causal association between childhood BMI and adult cardiometabolic traits

**Figure 2:**
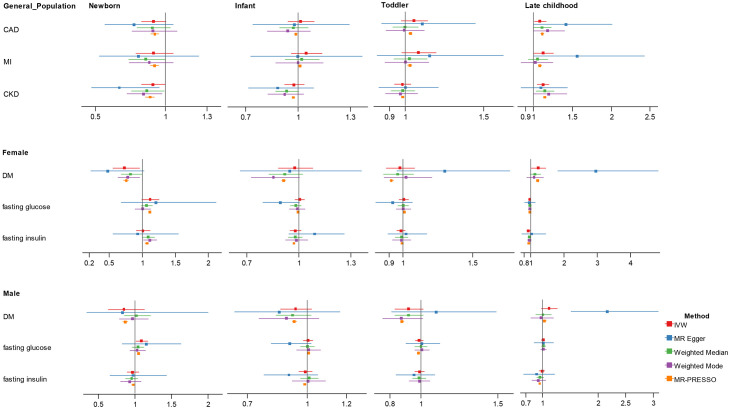
Association estimates of childhood body-mass index with adult cardiometabolic traits in univariate Mendelian randomization. Results show the odds ratio estimates and 95% confidence interval for BMI at different childhood stages. For DM, fasting glucose and fasting insulin with sex-specific GWAS data, results were shown in men and women separately; otherwise, results obtained in the total population were showed. Different colors represent methods used for Mendelian randomization analysis. CAD, coronary artery disease; MI, myocardial infarction; CKD, chronic kidney disease; DM, diabetes mellitus.

In univariate MR (**Figure 2**; **Supplementary Table 2**), we found suggestive evidence that increased childhood BMI at newborn was causally associated with reduced risk of DM for females (OR = 0.729, 95% CI 0.555-0.957, p = 0.023) and increased level of fasting glucose for males (OR = 1.092, 95% CI 1.016-1.174, p = 0.017). In late childhood, higher childhood BMI was a potential risk factor for CAD (OR = 1.083, 95% CI 1.010-1.162, p = 0.025), and MI (OR = 1.125, 95% CI 1.008-1.255, p = 0.036), and was significantly associated with CKD (OR = 1.122, 95% CI 1.046-1.203, p = 0.001) in total population regardless of sex. In this age group, we noticed suggestive causal association between BMI with increased risk of DM (OR = 1.217, 95% CI 1.020-1.452, p = 0.029), and lower fasting glucose (OR = 0.963, 95% CI 0.936-0.991, p = 0.009), as well as marginally significant association with fasting insulin (OR = 0.923, 95% CI 0.872-0.977, p = 0.006) in females, not in males. We did not identify significant association between infant or toddler BMI and adult cardiometabolic diseases or traits.

Heterogeneity and pleiotropy was detected in several analyses (**Supplementary Tables 3**, **4**). For those with significant associations in the primary analyses, using MR-Egger, weighted median, weighted mode and MR-PRESSO outlier-correction, we found the directions of the causal associations between childhood BMI and adult outcomes remained largely unchanged but with wider confidence interval, except that we observed stronger point effect estimate of five-to-eight-year BMI for DM in women (OR = 2.963, 95% CI 1.815-4.839, p = 0.001) in MR-Egger.

### 3.2 Multivariable Mendelian randomization on the causal effect of four stages childhood BMI on adult cardiometabolic diseases or traits

**Figure 3:**
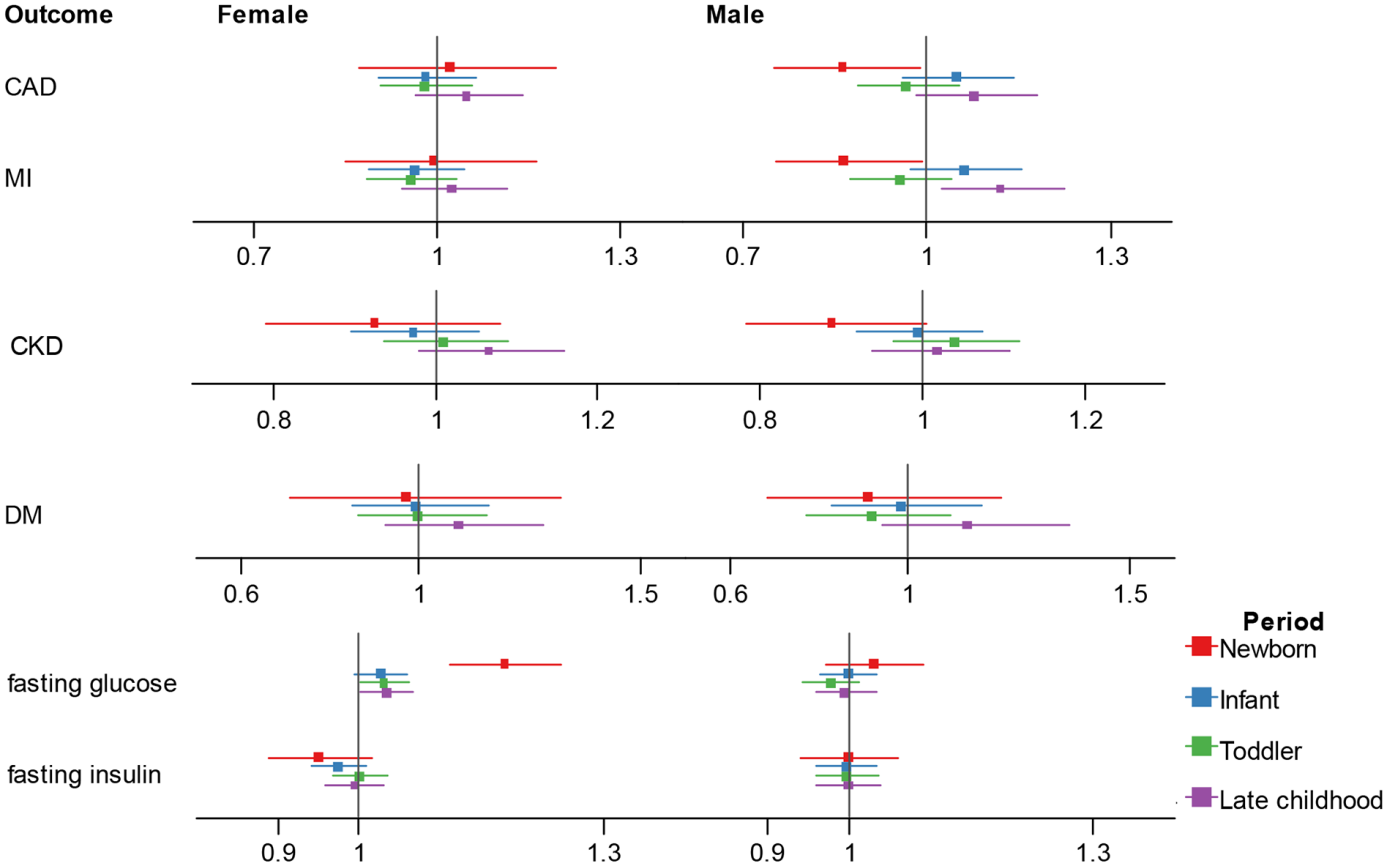
Association estimates of childhood body-mass index with adult cardiometabolic traits in multivariable Mendelian randomization. Results show the odds ratio estimates and 95% confidence interval for childhood BMI adjusting for adult female BMI and sex hormones, and male BMI and sex hormones. Different colors represent childhood stages. BMI, body-mass index; CAD, coronary artery disease; MI, myocardial infarction; CKD, chronic kidney disease; DM, diabetes mellitus.

In multivariable MR (**Figure 3**; **Supplementary Table 5**), in men, after adjusting for sex-specific IVs for adult BMI, testosterone and SHBG, there was a suggestive negative association between newborn BMI with CAD (OR = 0.862, 95% CI 0.751-0.989, p = 0.034) and MI (OR = 0.864, 95% CI 0.752-0.991, p = 0.037), while an increased effect size between five-to-eight-year childhood BMI and MI (OR = 1.120, 95% CI 1.023-1.226, p = 0.014).

In women, after adjusting for sex-specific IVs for adult BMI, SHBG and age at menarche, we observed independent effect of newborn, toddler and five-to-eight-year childhood BMI on fasting glucose (newborn: OR = 1.178, 95% CI 1.112-1.248, p = 2.76E-08; toddler: OR = 1.030, 95% CI 1.001-1.061, p = 0.044; five-to-eight-year childhood: OR = 1.033, 95% CI 1.001-1.067, p = 0.040), and the association direction was different from the univariate analysis for the five-to-eight-year group.

## 4 Discussion

In the present study, we observed age-specific impact of childhood BMI on adult cardiometabolic diseases/traits that newborn and late childhood BMI exerts larger impact than the infant and toddler ages. Besides, we found higher BMI at birth might be protective for several adult diseases but turned out to be a risk factor at late childhood. In addition, the causal associations between childhood obesity and adult outcomes greatly changed after adjusting for adult BMI and sex hormones. To the best of our knowledge, we are among the first to investigate the influence of age and sex on the associations between childhood obesity and adult outcomes, our study is therefore of novelty.

We noticed, despite with overlap of 95% CI of the effect estimates, childhood BMI tends to be associated higher risk of adult cardiometabolic diseases as the age increases. For instance, after adjusting for multiple potential covariates, we reported a positive association between the toddler- and five-to-eight-ye ar childhood BMI with CAD and MI. Meanwhile, it is noteworthy that, after adjusting for adult BMI, SHBG and testosterone, the effect of newborn BMI on CAD and MI, and the effect of five-to-eight-year childhood BMI on MI were statistically significant, that BMI at birth weight turned out to be a prospective factor. These causal associations revealed by this MR study were consistent with the findings in previous observational research, in which low BMI at birth was associated with later coronary events, while the positive associations between childhood BMI and the risk of adult diseases became stronger with increasing age (25, 26). Difference in results between newborn and late childhood might be attributed to two aspects. First, the beneficial effects of higher BMI at birth or the very early childhood on adult CAD might be caused by the negative influence of low birth weight, which can be marker for intrauterine growth restriction (26, 27). Second, several studies indicate that childhood weight development can be divided into different patterns, i.e., normal trajectory, early persistent obesity, early transient overweight and late rise delayed overweight (26); or by genetic predisposition, being classified into trajectory clusters of birth weight, early rise, transient and late rise (4), with varied impact on adult outcomes, thus, the BMI measured at certain time points may not represent a definite state of harmful obesity in childhood and, thus, do not have influences on health consequences in later life (28).

For CKD, we calculated the causal effect of childhood BMI on CKD incidence. Univariate MR suggested that only the five-to-eight-year childhood BMI had a positive effect on CKD. Similarly, a population-based cohort study reported the potential information that the early appearance of above-average BMI growth patterns provides in relation to adult-onset CKD (29). But adult BMI is still a strong risk factor for chronic kidney disease, after adjusting for adult BMI and sex hormones, Childhood BMI no longer had a significant effect on CKD.

Besides, we noticed a tendency that the magnitude of the impact of childhood BMI on certain cardiometabolic traits might be different. For instance, independent association between weight at newborn with CAD and weight at late childhood with MI was only observed in men, but not women; while the relationship between weight at newborn with adult fasting glucose was only significant in women. As previous studies have shown that childhood obesity is an important risk factor for DM in adults (30), the results of this study also show that there is a significant causal relationship between childhood BMI and DM. Univariate MR analysis showed that higher childhood BMI at five-to-eight-year childhood would increase the risk of DM in adults, especially for females, but the results were not statistically significant for males. Interestingly, at the time of birth, childhood BMI was inversely associated with DM in females, which suggested that higher childhood BMI in early years and higher childhood BMI in late years have quite different effect on adult diabetes. This may be due to the fact that diabetes risk is primarily associated with elevated BMI at the time of diagnosis (31). Moreover, a previous study indicated that childhood BMI trajectories have a significant impact on adult diabetes, independent of BMI levels, and the adolescence age period is a crucial window for the development of diabetes in later life (32). However, after adjusting for adult BMI and sex-related parameters, the effect of childhood BMI on DM was not statistically significant. Therefore, we consider that the effect of childhood BMI revealed in univariate MR was totally mediated by other potential confounding or mediators and has no independent effect on the adult disease.

There is a strong link between diabetes and glucose and insulin. Before the diagnosis of type 2 DM, insulin resistance may last several years, thus increasing insulin and glucose levels (33). We examined the causal relationship between childhood BMI with fasting glucose and fasting insulin by gender. For males, there was a positive causal relationship between newborn BMI and fasting glucose, consistent with observational findings. For females, five-to-eight-year childhood BMI was negatively associated with both fasting glucose and fasting insulin in univariate MR analyses, whereas multivariate MR results showed that childhood BMI had positive independent effect on fasting glucose after adjusting for adult BMI, SHBG and age at menarche except in infant.

There were still some limitations in this study. First, we assumed that the causal relationships between childhood BMI and all outcomes were linear. In fact, an observational study showed that some of these outcomes were nonlinearly related to childhood BMI (34). Second, the generalizability of the conclusion was limited since the GWAS data were mainly based on the European population. Third, sex-specific GWAS summary statistics for childhood BMI and certain outcomes were not publicly available and were not used in the current study. Despite that we adopted sex-specific IVs for the phenotypes with significant differences in genetic architecture in women and men, i.e., BMI, diabetes, fasting glucose, sex-related parameters, bias led by sex-specific exposures and sex-combined outcomes in MR cannot be fully ruled out and merits further updates. Lastly, residual and unmeasured confounding may still exist which requires further study. Accordingly, we would suggest future research to: firstly, conduct well-designed prospective cohort studies to evaluate the linear and nonlinear association between childhood BMI at different ages and cardiometabolic outcomes, extensively controlling for potential confounders or mediators; secondly, further explore the relationship between childhood BMI and cardiometabolic traits in men and women, and elucidate the potential mediating effects of sex hormones; and thirdly, explore the underlying mechanisms for the potential causality between age-specific childhood BMI and cardiometabolic outcomes.

In conclusion, our results suggest childhood BMI at different ages have differed causal associations with adult cardiometabolic traits. BMI at birth and late childhood may exert more significant impacts on adult cardiometabolic diseases than infant and toddler ages. These impacts of childhood BMI are influenced by sex disparities. The potential mechanisms for the causal associations between childhood BMI and adult cardiometabolic diseases are worth further investigation.

## Data availability statement

Publicly available datasets were analyzed in this study. This data can be found here: Summary statistics data for MR analyses have been contributed by the Norwegian Institute of Public Health (https://www.fhi.no/en/studies/moba/), the IEU GWAS database (https://gwas.mrcieu.ac.uk/), the Diabetes Genetics Replication and Meta-analysis Consortium (http://diagram-consortium.org/), the Meta-Analyses of Glucose and Insulin-related Traits Consortium (http://diagram-consortium.org), the Coronary Artery Disease Genome-wide Replication and Meta-analysis plus the Coronary Artery Disease Genetics Consortium (http://diagram-consortium.org/) and the CKDGen Consortium (https://ckdgen.imbi.uni-freiburg.de/).

## Author contributions

XZ and JY were responsible for the study’s concept and design. JY, YK, and XZ did the data cleaning and analysis. JY, CL, XY, MQ, PF, and XZ interpreted the data. JY and XZ drafted the manuscript. All authors contributed to the article and approved the submitted version.
